# Synthetic biology, metaphors and responsibility

**DOI:** 10.1186/s40504-017-0061-y

**Published:** 2017-08-29

**Authors:** Carmen McLeod, Brigitte Nerlich

**Affiliations:** 1 School of Geography and the Environment, University of Oxford, South Parks Road, Oxford, OX1 3QY UK; 2 School of Sociology and Social Policy, University of Nottingham, Nottingham, UK

## Abstract

Metaphors are not just decorative rhetorical devices that make speech pretty. They are fundamental tools for thinking about the world and acting on the world. The language we use to make a better world matters; words matter; metaphors matter. Words have consequences - ethical, social and legal ones, as well as political and economic ones. They need to be used ‘responsibly’. They also need to be studied carefully – this is what we want to do through this editorial and the related thematic collection. In the context of synthetic biology, natural and social scientists have become increasingly interested in metaphors, a wave of interest that we want to exploit and amplify. We want to build on emerging articles and books on synthetic biology, metaphors of life and the ethical and moral implications of such metaphors. This editorial provides a brief introduction to synthetic biology and responsible innovation, as well as a comprehensive review of literature on the social, cultural and ethical impacts of metaphor use in genomics and synthetic biology. Our aim is to stimulate an interdisciplinary and international discussion on the impact that metaphors can have on science, policy and publics in the context of synthetic biology.

## Introduction

During the twentieth century, the science of genetics grew exponentially in prominence. By the end of the millennium, historians and sociologists of science, as well as communication and media scholars, began to take stock of what had been achieved, publishing books with titles such as *The Meaning of the Gene* (Condit 1999) and *The Century of the Gene* (Keller 2009). In 2003, scientists successfully ‘read’ the full human genome for the first time and new types of bioscience began to emerge, including synthetic biology. Scientists working in this new field were hailed as being able to not only read, but to ‘write’ and rewrite genetic and genomic information. Related recent advances in ‘genome editing’ are accelerating developments in genomics and in synthetic biology.

These changes in knowledge and understanding of biological life are transforming and even blurring lines between ‘nature’ and ‘culture’. Anthropologist Stefan Helmreich observes how conceptions of the biological have become intertwined with the social:

“Biotechnology, biodiversity, bioprospecting, biosecurity, biotransfer, and other things *bio* – draw novel lines of property and protection around organisms and their elements (e.g. genes, organs), which now circulate in new ways as gifts, as commodities, and as tokens of social belonging or exclusion” (Helmreich 2016: 1).

Therefore, during the twentieth century, we have learned to speak a new biological language, which has influenced how we understand our bodies, our selves, and relations with the wider world. Over the last few decades, social scientists, linguists and synthetic biologists themselves have begun to discuss the new meanings of life and the associated hopes and fears that are emerging, leading to a rise in interdisciplinary work between the social and natural sciences.

Rather than confining this work to observing the research activities and the languages spoken in the context of synthetic biology, social researchers are also collaborating more directly with synthetic biologists to explore what it means to talk about doing scientific research ‘responsibly’ (see Nerlich and McLeod, 2016).

The **aim** of this thematic series is to stimulate discussion about how language shapes both emerging meanings of life in the context of synthetic biology and emerging meanings of responsibility. We will focus in particular on one potent cognitive and linguistic tool that enables humans to create new meaning, namely metaphor. Metaphors are not just decorative rhetorical devices that make speech pretty. They are fundamental linguistic and cognitive tools for thinking about the world and acting on the world (Lakoff and Johnson 1980). We will also consider how the science governance framework, Responsible Research and Innovation (RRI), and the notion of ‘responsibility’ are being mobilised through the use of metaphor within synthetic biology.

Researchers interested in RRI are keen to create a world in which research and innovation happen responsibly, taking the needs of society into account throughout the research process and beyond (see section on RRI below). In this context, the language we use to make a better world matters; words matter; metaphors matter. Words have consequences, even ethical, social and legal, as well as political and economic, consequences.

In this editorial, we wish to provide a launching point for considering the empirical and theoretical examples and concepts raised by the contributors to this thematic series. In the following, we shall first briefly summarise the history of the terms ‘synthetic biology’ and ‘responsible innovation’; we then provide an overview of research into metaphors in the context of genetics, genomics and synthetic biology and end with presenting some recent work on synthetic biology, metaphor and responsibility.

## A short genealogy of ‘synthetic biology’

The first to use the term synthetic biology (or ‘biologie synthétique’) was, most likely, the French biophysicist Stéphane-Armand Nicolas Leduc in 1912 (Peretó 2016). This was a time when the search for artificial life was the height of biological fashion and when scientists such as Jacques Loeb announced its creation (see Keller 2009; Morange, 2010a, b).

However, the nomenclature for the field of ‘synthetic biology’ is not straightforward. As Balmer and Martin pointed out in a first foray into synthetic biology and RRI, synthetic biologists have been debating their neologism for years (Balmer and Martin 2008). In a 2006 blog (now defunct), Rob Carlson, an early advocate of the subject, talked about the various labels for the new field, such as ‘Intentional Biology’, ‘Constructive Biology’, ‘Natural Engineering’, ‘Synthetic Genomics’ and ‘Biological Engineering’ (see Balmer and Martin 2008: 6). Quoting Evelyn Fox Keller (2002), Carlson (2010) suggests that, as the term ‘Synthetic Biology’ had been used for over a century, its continued use would be inevitable. This has proved to be the case. However, off and on fears are still voiced that the single word ‘synthetic’ connotes negative images of monstrous (unnatural) life forms let loose by maniacal scientists (see Roosth 2017).

While the name is old, the field in its modern form only emerged about fifteen years ago (see de Lorenzo and Danchin 2008). Around 2005 scientists at MIT, especially those working with Drew Endy (now Standford), started talking about a new discipline, which they called synthetic biology (Endy 2005; Andrianantoandro et al. 2006). Other leading figures shaping the field were (and still are) George Church at Harvard, Jay Keasling at the University of California and Craig Venter - the genomic entrepreneur and ‘father’ of the first synthetic cell in 2010. To this short list one can, of course, add many more names!

Modern synthetic biology has its roots in many fields, but is mainly based on a convergence of research in engineering, computing and modelling, with molecular biology, evolutionary genomics, and biotechnology on the one hand – and research into the origin of life, artificial life and orthogonal (parallel) life on the other (see Luisi 2006; Bedau et al. 2009; Peretó and Català 2007; Campos 2009). The current approach to synthetic biology also consists, predominantly, of three broad strands; DNA-based device construction, genome-driven cell engineering, and protocell creation (O’Malley et al. 2007). The Royal Academy of Engineering (2009: 6) describes the aims of synthetic biology as “…to design and engineer biologically based parts, novel devices and systems as well as redesigning existing, natural biological systems”.

Scientist Victor de Lorenzo (2008: 822) compares synthetic biology to a mixture of separate functioning biological components, not dissimilar to those created in human technologies. He explains that these components can “be described as a limited number of parts that can be combined in novel configurations to modify existing properties or to create new ones. In this context, engineering moves from being an analogy of the rational combination of genes—as in standard molecular biology and biotechnology—to becoming a veritable methodology with which to construct complex biological systems from first principles.”

Most importantly, those working within synthetic biology use genes, or rather standard DNA parts that encode basic biological functions, not only metaphorically, but literally as “the building blocks of life,” sometimes called “BioBricks” (Endy 2005; Biobricks 2016). This fusion between authentic (not metaphoric) engineering and molecular biology is an interesting phenomenon that will be further explored in this thematic series.

## A short genealogy of ‘responsible research and innovation’

In the 1970s, scientists engaged in recombinant DNA research instituted a form of ethical and social oversight in the form of the famous Asilomar conference^1^ and subsequent activities and publications. In the 1990s, genomics got its own form of social and ethical scrutiny in the form of a framework called ELSI (Ethical, Legal and Social Issues) in the US or ELSA (Ethical, Legal and Social Aspects) in Europe (see Zwart and Landeweert 2014). Synthetic biology now has its own official ethical framework in the form of ‘Responsible Research and Innovation’ (RRI). The aims of RRI are broader than both the Asilomar-type framework and ELSI and ELSA approaches, and it has emerged in a world where public understanding and public engagement activities are expected, as well as many other forms of science-society interactions fostered by scientists and policy makers since about the end of the 1980s (Short 2013).

Historically, the ‘RRI agenda began to emerge around 2010/2011 in a variety of shapes and forms, when a number of scholars started to write and blog about it such as René von Schomberg (2011), Jack Stilgoe (2011), Richard Owen, and Phil Macnaghten (Owen et al. 2012; Stilgoe et al. 2013). This new push for RRI has some of its roots in debates about the responsible use of emerging technologies, such as nanotechnology in around 2007, and reflections on how this fitted in with established frameworks of ethics, governance, public engagement and risk assessment (see Ribeiro et al. 2016).

In a very short time, RRI and its twin ‘responsible innovation’, became an important part of the European and UK funding and research scene. Like older enterprises, such as public engagement/dialogue/participation and so on, it has the support of both the scientific elite (funders and industry) and of those who see their task as critically engaging with science and technology from the perspective of science and technology studies. Interestingly, responsible innovation is now becoming itself an object of study (Randles 2013) for scholars in the fields of science and technology studies, the sociology of science, policy studies, anthropology and others.

There is also now a dedicated academic journal for the field: *Journal of Responsible Innovation*. Since around the start of the millennium, research proposals submitted to physical, engineering, biological and medical science funders in the UK (the EPSRC and the BBSRC) have been required to include a section in which researchers explore how their research engages with the wider public sphere, how it might lead social and economic impact in the wider world, and how it intends to implement RRI.

Definitions of RRI vary. The Engineering and Physical Sciences Research Council defines RRI as “a process that seeks to promote creativity and opportunities for science and innovation that are socially desirable and undertaken in the public interest” (Engineering and Physical Sciences Research Council, 2017). The leading architect of RRI in the EU context, René von Schomberg, defines RRI as: “…a transparent, interactive process by which societal actors and innovators become mutually responsive to each other with a view to the (ethical) acceptability, sustainability and societal desirability of the innovation process and its marketable products (in order to allow a proper embedding of scientific and technological advances in our society)” (von Schomberg 2011). There are further definitions, used especially in Europe, which also encompass specific concerns, such as open access, gender equality and science education (European Commission 2017). All versions of RRI emphasise the importance of societal involvement in science and technology innovation from the start of a research project (‘upstream’) and throughout its life-time.

Funders hope that, through RRI, innovations can happen on a more socially responsible and responsive basis and, at the same time, steer innovations towards the right impacts in an ethical and democratic way. Funders also expect that the innovation process can meet these goals while staying economically competitive despite the broader financial and socio-economic challenges that societies are grappling with. In the UK, policy makers are even hopeful that the combination of RRI and synthetic biology through publically funded Synthetic Biology Research Centres, will bring such positive economic outcomes as to assuage some of these socio-economic problems (Synthetic Biology Leadership Council, 2016).

In the context of synthetic biology, RRI is becoming part of a new language spoken between two academic communities, namely natural and social scientists (Balmer et al. 2016). Whether members of the public beyond academia ‘speak’ RRI is doubtful. For example, the current entry for RRI within Wikipedia carries a warning that the text may need improving, with the specific comment: “perhaps somebody could translate this gobbledygook (Euromanagerspeak)” (Wikipedia.org 2017). Some might even argue that RRI could be categorised as a ‘buzzword’.

Philosopher and historian of science, Bernadette Bensaude Vincent (2014), has analysed the politics of buzzwords focusing in particular on the concept of ‘public engagement’. She also mentions a number of other buzzwords, such as ‘responsible innovation’. She points out that buzzwords have their roots in marketing and are sometimes defined …as “hollow terms, with more hype than substance” (p. 240), or as the online edition of the *Oxford English Dictionary* puts it: “a term used more to impress than to inform” (OED online n/d). Could it be the case that responsible innovation is such a term? But if it is hollow and hype why does RRI attract so much attention and so many proponents? Bensaude Vincent argues that terms such as ‘public engagement’ and ‘responsible innovation’ are ‘value-laden’, and not meant to be ‘revolutionary’ but rather to actively help smooth changes in values in society. In particular, she argues that these types of words should be seen as symptomatic of “the inextricable connection between science, technology, society and economics in the current regime of research and development” (Bensaude Vincent 2014: 250).

Responsible innovation comes as part of a cluster of phrases, which all reinforce each other. These are: responsible innovation, sustainable development and, of course, public engagement. Together they convey a message that is easily remembered, albeit vague. Such buzzwords are especially potent when they appear in times of crisis and seem to show a way out of the crisis. In the case of the 2008 global financial crisis, Bensaude Vincent suggests new ‘miracle’ technologies like synthetic biology seem to show a ‘responsible’ economic path leading beyond austerity and towards wealth creation. She also notes that buzzwords spread, like rumour, from mouth to mouth, paper to paper, institution to institution. In the case of responsible innovation this happened through academic papers, blogs, briefing documents and, most importantly, ‘frameworks’ for and by funders both in the UK and in Europe, and now also in the US. Once widely spread, buzzwords establish something like a ‘trading zone’ (see Murphy et al. 2016) in which people from different backgrounds, such as funders, natural and social scientists, policy makers and industrialists, can communicate without however having to be too explicit about what they are saying. Responsible innovation becomes something of a metaphor.

The success of responsible innovation or RRI as a buzzword and metaphor, the speed with which it has spread and established itself, is quite astounding. Another reason for this, apart from the flexible way with which it can be used, may be that it links up with and reinforces prominent cultural values and also promises to enable a way of innovating and creating wealth without destroying such values. RRI promises to deliver innovations that are ethically acceptable, safe, sustainable and socially desirable.

In this way, RRI creates expectations that ‘mobilise the future into the present’ (Brown and Michael 2003), while at the same time trying to anticipate and assess the impacts that possible futures may have on the present. This is a complex task that needs more scrutiny than it has so far received, and not only in academic circles. We might need a responsible innovation approach to RRI itself, including the use of the phrases/acronyms ‘responsible innovation’ or RRI as quasi-magical words bringing forth changes in research culture and public participation with research. The term ‘synthetic biology’ itself and the metaphors used in the field and those promoting the field also create expectations about the future, which need equal monitoring.

## Metaphors and synthetic biology

The interaction between language, science and society has fascinated social and cultural scientists, anthropologists and linguists for many years, in particular with respect to the history of the biosciences. Both natural scientists active in the field, and social scientists observing this work, became fascinated by the role of metaphor in the articulation of scientific concepts on the one hand and articulating the science for wider society on the other (Turney 1998; Avise 2001). Metaphor analysts also began to scrutinise metaphors used to either ‘sell’ science or to ‘shape’ public attitudes (Nelkin and Lindee 1995), as well as their use by journalists who broker knowledge between science and society (see Maasen and Weingart 2000; Nerlich et al. 2004; Nerlich et al. 2005; Nerlich et al. 2009).

Surprisingly, as the life-sciences, especially genetics, advanced from early genetics to genomics, to post-genomics – and from cloning research to stem cell research and beyond – the metaphors used in science and society seemed to stay quite stable, with only slight variations around the fringes (Turney 2005; Nerlich and Hellsten 2004; Hellsten and Nerlich 2008; Zwart 2009a, 2009b; 2010). For example, for a long time, there have been references to blueprints, maps, programmes and books, especially the ‘book of life’ and the reading, deciphering, decoding of this book.

These metaphors can be read in quite deterministic ways and have led to some social scientists expressing concerns that increased knowledge of human biology will lead to an increasingly reductionistic worldview where moral and ethical frameworks will be founded increasingly on biological attributes (see Kaye 1997). However, as research by Celeste Condit has shown, this might just be an impression, rather than a reality (see Condit et al. 1998). This does not mean, however, that social scientists have not continued to voice such concerns as genetic and genomic research advanced over time.

Things appeared to shift with the advent of research into the ‘microbiome’ (the totality of microbes, their genetic elements, genomes, and environmental interactions in a defined environment, e.g. the human gut). In this context, what was seen as deterministic discourses seemed to begin to break down as issues around complexity, communities and context were foregrounded (see Nerlich and Hellsten 2009). Genes and bacteria interactions began to be investigated and popularised (Turney 2015; Yong 2016). It became apparent that we are more than just our genes and genomes; that we interact constantly, indeed we are co-constituted by our interactions with bacteria and environments.

Similarly, epigenetics has attracted the attention of metaphor sleuths (Stelmach and Nerlich 2015). This was especially interesting, as epigenetics is not yet a settled field and metaphors are not settled either; there is however a lot of hype. But whatever the hype, epigenetics has focused the scientific lens more on gene-environment interactions and stimulated public debate about such interactions and their social and political implications, which can only be a good thing.

Together with microbiomics and epigenetics, synthetic biology contributed to making us think, yet again, about the meaning of life and of what makes us human. Around 2010, claims began to be popularised that scientists were not only able to ‘read’ the ‘book of life’ (and ‘see’ who we are), but were now also able to ‘write’ it and ‘edit’ it. Synthetic biologists claimed that they could do even do more than ‘just’ writing; that they could create, construct, indeed, ‘engineer’ or ‘design’ ‘artificial life’ (Cserer and Seiringer 2009; Hellsten and Nerlich 2011).

In this context quite narrowly mechanistic metaphors emerged; for example: ‘an organism is a machine’, ‘an organism is a factory’, ‘an organism is a computer’ or ‘an organism is a chassis’, ‘metabolic pathways are electronic circuits’. Advances in sequencing technologies as well as gene editing technologies, such as CRISPR/Cas9, mean that scientists in institutions, as well as DIY enthusiasts, can ‘cut and paste’ or ‘edit’ genes in and out of (human, animal, plant, bacterial etc.) genomes relatively easily; as well as turning or switching genes on and off (a metaphor that also pervades epigenetics and the study of gene expression and regulation).

With these metaphors we enter a metaphorical field governed by a different master metaphor compared to the older ‘book of life’ metaphors. One might call it the ‘circuit of life’ metaphor. This metaphor (which links up with the older one of ‘programming life’) shifts the way we talk and think about genes and genomes away from the book (and cutting and pasting and editing paper) and towards the machine and the computer. Circuit and machine metaphors have come to dominate thinking and talking about synthetic biology, which has, indeed, been defined as the “application of rigorous engineering principles to biological system design and development” (Royal Academy of Engineering 2009: 5). However, there have been some critiques of machine metaphors used in synthetic biology.

In their article “The mismeasure of machine: Synthetic biology and the trouble with engineering metaphors”, Boudry and Pigliucci (2013: 667) suggest that the use of metaphors in the complex and messy world of biology may do some harm. While acknowledging that the use of analogy and metaphors are important ways that humans make sense of highly specialised aspects of society, they argue that “…it may simply be the case that the object of study becomes so remote from everyday experience that analogies begin to do more harm than good.”

Porcar and Peretó (2016) go even further in their critique of the ‘organism is a machine’ metaphor. Their analysis of the differences between machine technologies created by humans and biological systems, leads them to the unequivocal conclusion that “…cells are not machines, which has important theoretical and practical implications for the current development of SB. We suggest that further progress within the SB framework will be achieved by abandoning the bio-machine paradigm and by using an alliance between engineering and evolution as a guiding tool” (Porcar and Peretó 2016: 451).

Alongside ‘serious’ engineering, machine and design metaphors, synthetic biologists also use a language of play and fun. Where formerly they spoke of the ‘building blocks of life’, they now speak of ‘biobricks’ and compare them to Lego, Erector sets and Lincoln Logs (Roosth 2017: 25). They also use words like ‘tinkering’, ‘sewing’, ‘stitching’.

Overall though, synthetic biology seems to be fundamentally grounded in three ‘big’ metaphors (Hellsten and Nerlich 2011), namely ‘organisms are books’ that can be read, edited and written); ‘organisms are engines or machines’ that can produce ‘stuff’; and ‘organisms are computers’ that can be programmed to do things. These metaphors are the promissory backdrop to grand synthetic biology discourses, while the ‘small’ metaphors of tinkering and play distract, to some extent, from the deterministic and somewhat intimidating flavour of these big metaphors.

The three big metaphors are linked to three big technological ‘revolutions’: the printing revolution initiated by Gutenberg in the 1400s; the industrial revolution grounded in new types of engines, engineering and machines that started in the 1800s and bringing with it standardised parts, mass production and assembly lines, and the computer or information revolution that began in the mid-1900s. In a sense, synthetic biology is framed as partaking in all three revolutionary processes and to be highly revolutionary in turn. It is often referred to as being a key part of the fourth industrial revolution, providing the innovatory power to “customize organisms by writing DNA” (Schwab 2016: 21).

Metaphors of books, machines and computers are all highly visible in debates about synthetic biology. They frame discussions about life and the living in terms of reading/writing/editing, designing/engineering and mass production, thus emphasising the power, but not really the responsibility, of science and scientists. This power is now doubly asserted as ‘editing’ has moved from being a mere metaphor to being a ‘reality’ in the form of ‘gene editing’ – and thus needs to take place responsibly, given that mistakes are as easy to make as the technology is to use.

O’Keefe et al. (2015) were the first to look systematically into the role of metaphors in shaping the emerging public meaning of gene editing and CRISPR, by investigating the use of metaphors in American newspapers and popular science publications. The overarching metaphors they found were both old and new ones. The old ‘blueprint‘ metaphor, which has been used for the human genome for decades, is still in use, as well as the ‘code’ and ‘map’ metaphors. Newer ones are ‘gambling’, ‘mechanism’, ‘medicine’ and ‘origami’. ‘War and fight’ metaphors were found as well, and under the overarching metaphor of ‘medicine’, they detected talk of ‘scalpel’, ‘surgery’, ‘snipping’, amongst others.

O’Keefe and her colleagues also found that the most common metaphor in use is that of the genome as ‘text’ and that the idea of ‘editing’ appears in nearly every article. Not surprisingly, there are also references to ‘cutting and pasting’ and ‘scissors’. A new metaphor used in the articles they studied is that of ‘targeting’ (a rather popular metaphor in the discourse of nanomedicine), used “both to emphasize precision and to warn of the dangers of unintended cuts”, that is, ‘off-target’ mistakes in the editing process (p. 8). The article concludes that “although CRISPR metaphors are not settled, the metaphors that are gaining traction obscure and mislead in important ways”, in particular conveying a level of precision that has not yet been reached (O’Keefe et al. 2015: 8). Like Avise (2001), who analysed metaphors at the height of the human genome project, the authors speculate about whether it is possible to find better metaphors, such as ecological ones, that might capture the complexity of interfering with genes and genome more accurately.

O’Keefe et al.’s (2015) article provoked a response from Nelson et al. (2015: 61) who called for CRISPR metaphors to be disentangled more carefully, particularly highlighting the need for distinguishing “…between metaphors for what CRISPR *is*, as a technology, versus what CRISPR *does*, in applications”. Only such careful analysis can reveal in which ways CRISPR metaphors may illuminate public discourse or obscure it.

## Metaphors, ethics and responsibility

There seems to be quite a wave of interest in the ways that metaphors frame synthetic biology and in exploring the ethical, legal and social implications of such framings – and for good reason. Richard Jones, a soft matter physicist and policy expert, once said in 2010, at a time when interest in synthetic biology first peaked in science and society: “How much do we need to worry about a few arguable metaphors? Here, more than usually, because it is these ideas of complete control and the reduction of biology to the digital domain that are so central in investing the visions of synthetic biology with such power” (Jones, 2010).

While early metaphors framed synthetic biology in the media as a powerful science, strangely devoid of responsibility, it should be stressed that synthetic biologists quite commonly reflected on their responsibilities to society from the very beginning. They established voluntary codes of ethics (Check 2006; BIOSINT, 2015), established an open registry of standard biological parts (iGEM 2017; Galdzicki et al. 2014) as early as 2005.

A further example of synthetic biologists concerns about responsibility relates to the emergence of CRISPR-Cas9 technology around 2015. At this time, a new Asilomar conference was convened with a follow-up article published in *Science* calling for caution (Baltimore, 2015). *Nature* (2015) also published a special issue on CRISPR which included articles relating to concerns about the governance and biosafety of this new gene editing technique.

Chinese researchers who used CRISPR to experiment for the first time on a human embryo in April 2015 made their (mainly negative) results public, a decision that was described as ‘ethical’ and ‘could reduce both risky and pointless research’ (Sandberg 2015). The Chinese research was published in the online journal *Protein & Cell* whose editor then wrote an editorial defending the decision to publish, also calling for restraint and ethical, social and legal reflection: “Until a consensus on new regulatory rules can be reached, it is in the best interest of all parties that the research field should voluntarily avoid any study that may pose potential safety and/or ethical risks. Only by holding themselves to the highest standards will scientists retain the public’s trust in biomedical research, and at the same time, provide the best service for the well-being of our society (Zhang 2015: 313).”

All these developments are, of course, scrutinised by social scientists, who have been observing them for about a decade or more. From about 2006 onwards a growing number of social scientists and bioethicists began to immerse themselves in exploring the relationship between synthetic biology and issues related to broader societal concerns such as ethics public engagement/deliberation and risk (e.g. O’Malley et al. 2007; van Est et al. 2007; Balmer & Martin 2008; Calvert 2008; Yearley 2009; Lentzos et al. 2009; van den Belt 2009; Keller 2009; Schmidt et al. 2009; Rabinow and Bennett 2012).

However, social science and humanities scholars have, so far, paid little attention to the language used to establish the field and to popularise it and explored the ethical implications of that language use. Some work has been published since 2009, such as a chapter considering Craig Venter’s work in relation to media presentations of metaphor use and ethical discourses of synthetic biology (Balmer and Herremann 2009) and an article on metaphors used in ‘artificial life reporting’ by Hellsten and Nerlich (2011). In 2011, a synthetic biologist published an article entitled “Beware of metaphors: chasses and orthogonality in synthetic biology” (de Lorenzo 2011). In 2012 Marianne Schark (2012) published an article denouncing the machine metaphor in synthetic biology and in 2013 the STS scholar Pauwels (2013) published a commentary piece in *Nature* entitled: “Communication: Mind the metaphor” warning of the damage that might be done by engineering metaphors. Interestingly, responsible use of language in science more generally also began to be discussed for example by Brendon Larson in the context of environmental science (Larson 2011; Kueffer and Larson 2014).

In recent years, especially in Germany, some books have been published dealing more directly with metaphors, ethics and responsibility. For example, in 2016, an edited collection entitled *Genetic Transparency: Ethical and Social Implications of Next Generation Human Genomics and Genetic Medicine* appeared (Dreyer et al. eds. 2016). The book focuses on questions about who should have access to information relating to personal genomics, but also highlights that “the social and cultural meanings of DNA and genetic sequences are much richer than can be accounted for by purely biomedical knowledge” (Dreyer et al. 2016).

Another book, also published in 2016, is more specifically focused on metaphor. It is entitled *Synthetic Biology: Metaphors, Worldviews, Ethics and Law* (Boldt, 2016a) and aims to assess social, ethical, and philosophical perspectives on synthetic biology beyond purely assessing potential risks and benefits of its applications. The volume also discusses potential challenges relating to governance and regulation. A chapter by (Boldt, 2016b) highlights some of the issues that will elaborated further in this thematic series. In particular, he argues that by literally or figuratively turning nature into a ‘tool’, we risk missing “important development properties of living beings and hinder the evolution of many sources of unexpected value. This is not what synthetic biology need or ought to be about” (Boldt, 2016b: 8)

A further book dealing with language, responsibility and synthetic biology is entitled *Ambivalences of Creating Life: Societal and Philosophical Dimensions of Synthetic Biology* (Hagen et al. eds. 2016). This edited collection contains a fascinating chapter by Daniel Falkner (2016), based in his PhD thesis, which overlaps with some of the topics tackled in our thematic series focus. For example, Falkner emphasises the key, but often overlooked role that metaphors play in the life sciences and especially novel biotechnologies. He argues that current discussions about synthetic biology provide important exemplars of the ways in which metaphors become entangled with debates about new technologies and the place of science and ethics. Falkner observes “there seems to be a connection between the paradigm shift in the epistemological approach, the technological development, the societal discourse and the metaphors that have been used to describe, explain and argue the new field of synthetic biology and its revolutionary nature.” (Falkner 2016: 252). The analysis focuses in particular on the metaphor of the ‘genetic code’ and the ways in which this has become a reference point in narratives that describe the evolution of synthetic biology from key figures, such as Erwin Schrödinger to Craig Venter.

## Conclusion

We argue that it is important to think about metaphors because they are not only used to *explain the world*, but they also affect how we *think about the world*, they structure “…our attitudes about public – and scientific – issues” (Nelkin 2001: 556) and they influence how we act upon and shape the world we live in. We agree with Martin Döring (2014) that it is surprising that, as the use of metaphors in relation to biotechnology innovation has intensified, there is still a lack of systematic study of the normative implications, and associated moral and ethical assumptions, inherent in this metaphors use. In this thematic series, we aim to address this lack.

## Footnotes

1 The Asilomar Conference on Recombinant DNA was held in February 1975 at Asilomar State Beach near Monterey in California, US. The conference was organized by scientist, Paul Berg, with the goal of discussing potential ‘biohazards’ of research relating to recombinant DNA, with the particular aim of exploring whether regulatory restraints at that time were appropriate (see Berg et al. 1975).
